# Salubrinal in Combination With 4E1RCat Synergistically Impairs Melanoma Development by Disrupting the Protein Synthetic Machinery

**DOI:** 10.3389/fonc.2020.00834

**Published:** 2020-06-19

**Authors:** Gregory R. Kardos, Raghavendra Gowda, Saketh Sriram Dinavahi, Scot Kimball, Gavin P. Robertson

**Affiliations:** ^1^Department of Pharmacology, The Pennsylvania State University College of Medicine, Hershey, PA, United States; ^2^The Melanoma and Skin Cancer Center, The Pennsylvania State University College of Medicine, Hershey, PA, United States; ^3^The Melanoma Therapeutics Program, The Pennsylvania State University College of Medicine, Hershey, PA, United States; ^4^Department of Cellular and Molecular Physiology, The Pennsylvania State University College of Medicine, Hershey, PA, United States; ^5^Department of Surgery, The Pennsylvania State University College of Medicine, Hershey, PA, United States; ^6^Department of Pathology, The Pennsylvania State University College of Medicine, Hershey, PA, United States; ^7^Department of Dermatology, The Pennsylvania State University College of Medicine, Hershey, PA, United States

**Keywords:** ALDH18A1, protein synthesis, cyclins, eIF2α, targeted therapy

## Abstract

Increased protein synthesis is a key process in melanoma, which is regulated by the ALDH18A1 gene encoding pyrroline-5-carboxylate synthase (P5CS). P5CS is involved in proline biosynthesis and targeting ALDH18A1 has previously been shown to inhibit melanoma development by decreasing intracellular proline levels to increase the phosphorylation of eIF2α mediated by GCN2, which then impairs mRNA translation. Since there are no current inhibitors of P5CS, decreased eIF2α phosphorylation in melanoma was targeted using salubrinal (a specific inhibitor of eIF2α phosphatase enzymes). While salubrinal alone was ineffective, the combined use of salubrinal and 4E1RCat (a dual inhibitor of eIF4E:4E-BP1 and eIF4E:eIF4G interaction to prevent assembly of the eIF4F complex and inhibit cap-dependent translation) was found to be effective at decreasing protein synthesis, protein translation, and cell cycle progression to synergistically decrease melanoma cell viability and inhibited xenograft melanoma tumor development. The combination of these agents synergistically decreased melanoma cell viability while having minimal effect on normal cells. This is the first report demonstrating that it is possible to inhibit melanoma viability by targeting eIF2α signaling using salubrinal and 4E1RCat to disrupt assembly of the eIF4F complex.

## Introduction

Advancements in our understanding of dysregulated signaling pathways in melanoma have resulted in the development of drugs targeting pathway alterations, such as BRAF and MEK1/2 inhibitors. Although these inhibitors have improved patient care, resistant disease eventually develops. This resistance can occur through secondary alterations, which re-activate signaling pathways (1–3). As melanoma is capable of pathway re-activation, it is important to identify therapies that target downstream of these signaling pathways.

A major component of melanoma development is the constitutive activation of the MAPK pathway, predominantly resulting from BRAF or NRAS mutations (4, 5), which contributes to increased cell proliferation and survival. In addition, the PI3K/AKT pathway is also activated in many melanoma cases, which can impair apoptosis and promote cell proliferation (6, 7). These pathways converge to increase protein production, by regulating translational initiation (8–11). One study has reported that resistance to anti-BRAF, anti-MEK, and combination treatments was mediated by formation of the eIF4F translation initiation complex in melanoma, colon, and thyroid cancer cell lines (11). Inhibition of the eIF4F complex was reported to synergize with mutant BRAF V600E inhibition (11). Therefore, translation initiation signaling has been identified as a promising pathway for therapeutic targeting.

We have previously reported that targeting proline biosynthesis, through siRNA knockdown of ALDH18A1, is capable of significantly inhibiting melanoma cell and xenograft tumor growth (12). The ALDH18A1 gene encodes pyrroline-5-carboxylate synthase (P5CS), which is involved in proline biosynthesis. In melanoma, P5CS knockdown decreased intracellular proline levels, which increased GCN2 and eIF2α phosphorylation (12). GCN2 is a kinase of eIF2α that senses nutrient starvation (13), and phosphorylation of eIF2α impairs mRNA translation (14, 15). Since there are no current inhibitors of P5CS, the efficacy of agents targeting the protein synthetic machinery through this pathway was evaluated. Salubrinal (Sal) inhibits eIF2α dephosphorylation by disrupting GADD34- and constitutive repressor of eIF2α phosphorylation (CReP)-induced recruitment of eIF2α to protein phosphatase 1 (PP1) (16). An increase in phosphorylated eIF2α impairs translation initiation by inhibiting the guanine nucleotide exchange factor (GEF) activity of eIF2B (17). Inhibition of eIF2α phosphorylation has been reported to induce transformation in a fibroblast cell model (18), and inactivation of PTEN in melanoma was reported to decrease eIF2α phosphorylation (19). Thus, the ability of Sal to increase eIF2α phosphorylation may be a useful approach to disrupt melanoma development.

Another agent, 4E1RCat, impairs eIF4F assembly through its binding to eIF4E, inhibiting cap-dependent translation (20). Transformation via the PI3K and AKT pathways have been reported to involve eIF4E activation (21). In addition, the MAPK pathway, through MNK1/2 phosphorylation, activates eIF4E, promoting transformation (22, 23). Therefore, 4E1RCat targets downstream of signaling pathways important for melanoma development.

As there are no current inhibitors of P5CS, the protein product of ALDH18A1, we proposed to target the ALDH18A1 pathway by inhibiting eIF2α/β, but inhibition by Sal alone was ineffective. Therefore, we screened several inhibitors of protein synthetic machinery signaling, and identified 4E1RCat as a potential agent that synergizes with Sal. Sal in combination with 4E1RCat was found to inhibit xenograft melanoma tumor development and synergistically decreased melanoma cell viability. This combination decreased the protein synthetic machinery and cell cycle progression. Thus, targeting the protein production with Sal and 4E1RCat effectively impaired melanoma viability and could be used to target melanoma cells having high levels of ALDH18A1.

## Materials and Methods

### Cell Line and Culture Conditions

The normal human fibroblast cell line FF2441 (provided by the laboratory of Dr. Craig Myers, Penn State College of Medicine, Hershey, PA) was maintained in DMEM (Thermo HyClone, Logan, UT, USA) supplemented with 10% FBS (Thermo) supplemented with 1 × GlutaMAX (Life Technologies, Carlsbad, CA). Melanoma cell lines UACC 903 (containing BRAF V600E; provided by Mark Nelson, University of Arizona, Tucson, AZ), 1205 Lu (containing BRAF V600E; provided by Dr. Meenhard Herlyn, Wistar Institute, Philadelphia, PA), A375M (containing BRAF V600E; CRL-1619; ATCC, Manassas, VA), C8161.Cl9 (lacking BRAF V600E; provided by Dr. Danny Welch, University of Kansas, Kansas City, KS), and MelJuSo (lacking BRAF V600E; provided by Dr. Judith Johnson, University of Munich) were grown in DMEM with 10% FBS and 1 × GlutaMAX. All cell lines were maintained in a humidified incubator at 37°C and 5% CO_2_ atmosphere and periodically monitored via phenotype, genetic biomarkers, and growth potential to confirm identity.

### Determination of Cell Viability

Cell viability was measured using the 3-(4,5-dimethylthiazol-2-yl)-5-(3-carboxymethoxyphenyl)-2-(4-sulfophenyl)-2H-tetrazolium (MTS) assay (Promega, Madison, WI). To test the effects of drug treatment on cell viability, varying concentrations of Sal (Sigma, Adooq) and 4E1RCat (Sigma, MedChem express) were dissolved in DMSO and added to cells. To determine the optimal concentrations, each was tested individually with concentrations ranging from 5 to 50 μM. Optimized concentrations were used for *in vivo* experiments. Cells were treated with Sal, 4E1RCat, the combinations, or DMSO control at indicated concentrations for 72 h, followed by addition of MTS for 1 h and absorbance was measured at 490 nm, using Soft Max Pro software. Synergy analysis was calculated as described previously according to the Chou-Talalay method using the Calcusyn software (24, 25). Combination index (CI) values < 0.9 were synergistic, 0.9–1.1 were additive, and >1.1 were antagonistic.

### Western Blotting

Lysates were prepared and electrophoresed on gels as described previously (26). Membranes were probed with primary antibodies following each of the supplier's recommendations. Cyclin A2, Cyclin B1, Cyclin D1, Cyclin E1, Cyclin E2, Cyclin H, CDK2, ERK2, and the secondary antibodies were purchased from Santa Cruz. The immunoblots were developed using ECL Western Blotting Substrate (Thermo Scientific) or Supersignal West Femto Chemiluminescent Substrate (Thermo Scientific).

### Animal Studies

Animal experiments to assess the efficacy of drug treatment were performed according to the protocol approved by the Institutional Animal Care and Use Committee at Penn State University. Subcutaneous injection of 1 × 10^6^ UACC 903 or 1,205 Lu melanoma cells were injected above both the left and right rib cages of 4–6-week-old female Athymic-Foxn1nu nude mice (Harlan Sprague Dawley Inc.) (27). Six to eight days later, when a fully vascularized tumor had formed, mice were randomly divided into a vehicle and experimental groups. The ratio of Sal to 4E1RCat for experimentation was based on the published literature. Specifically, Sal at 1 mg/kg has been used by multiple groups for its ability to inhibit tumor xenografts (28) and other pharmacological activities (29–31). However, 4E1RCat was not well-studied in mouse models, and hence, the dose of Sal was fixed and 4E1RCat varied to assess and maximize the combined effect.

In UACC 903 xenograft experimentation, multiple doses of 4E1RCat (2.5–15 mg/kg) along with 1.0 mg/kg body weight of Sal were tested: in brief, Group 1 (DMSO vehicle control), Group 2 (Sal, 1.0 mg/kg), Group 3 (4E1RCat, 15.0 mg/kg), Group 4 (Sal, 1.0 and 4E1RCat 2.5 mg/kg), Group 5 (Sal, 1.0 and 4E1RCat 5.0 mg/kg), Group 6 (Sal, 1.0 and 4E1RCat 10 mg/kg), and Group 7 (Sal, 1.0 and 4E1RCat 15 mg/kg). Subsequent validation studies were conducted with 1,205 Lu melanoma cells and the selected drug combination of 1.0 mg/kg Sal and 4E1RCat 10 mg/kg as well as the controls. All drugs were administered intraperitoneally on alternate days for 3–4 weeks (4 mice/group; 2 tumors/mouse). Body weight in grams and dimensions of developing tumors in cubic millimeters were measured on alternate days.

### Toxicity Assessments

At the end of tumorigenicity assessment, blood was collected from each euthanized animal in a serum separator tube with lithium heparin (BD Microtainer) following cardiac puncture, and subjected to a routine available panel for assessing major organ-related toxicity (24, 25, 32, 33). Levels of GLU (Glucose), BUN (Blood urea nitrogen), CREA (Creatinine), CAL (Calcium), TPR (Total Protein), ALB (Albumin), GLB (Globulin), ALT (Alanine aminotransferase), ALKP (Alkaline phosphatase), TBIL (Total bilirubin), and AMY (Amylase) were assessed as a part of the panel.

### Cell Cycle Analysis

Two days after drug treatment, 1 × 10^6^ asynchronously proliferating cells were collected per treatment. Harvested cell pellets were washed twice with serum-free DMEM followed by resuspension in propidium iodide staining solution (0.1 mg/ml propidium iodide, 0.02 mg/ml Ribonuclease A, 1 mg/ml sodium citrate, and 0.3% Triton-X-100 in 1 × PBS). Cells were analyzed using a BD FACSCalibur (BD Biosciences, San Jose, California, USA) and results were analyzed by ModFit LT (Verity Software House, Topsham, Maine).

### Protein Synthesis Analysis

Protein synthesis analysis was performed as described previously using the Click-iT Protein Reaction assay (Life Tech) (26). A total of 100,000 melanoma cells were plated in P100 plates and treated with the protein synthesis inhibitors. Two days post treatment with Sal, 4E1RCat, or combination, cells were starved of methionine by washing plates with PBS and then incubated with methionine-free DMEM for 1 h. Respective plates were maintained with Sal and/or 4E1RCat in methionine-free DMEM. Cystine and methionine-free DMEM (Life Technologies, 21013–024) supplemented with 48 mg/L L-cystine (#2470, CalBioChem) was used for methionine starvation. Cells were subsequently incubated with azidohomoalanine (AHA) for 4 h at 37°C. Protein lysates were collected using RIPA buffer and proceeded to protein synthesis analysis according to the manufacturer's instructions.

### Polysome Analysis

Cells were seeded in 150-mm culture dishes and grown up to 70% confluence (~1.0 × 10^7^ cells). Following the indicated treatment as described, cycloheximide (CHX) (100 μg/ml) was added to cells for 10 min at 37°C. Cells were washed twice with cold PBS containing CHX (100 μg/ml), scraped off, and pelleted at 4,000 rpm for 10 min. The cell pellets were suspended in 500 μl of lysis buffer [10 mM HEPES-KOH at pH 7.4, 2.5 mM MgCl_2_, 100 mM KCl, 0.25% NP-40, 100 μg/ml CHX, 1 mM DTT, 200 U/ml RNase inhibitor (RNaseOUT, Invitrogen), and EDTA-free protease inhibitor]. Lysates were cleared at 14,000 rpm for 15 min and supernatants (cytosolic cell extracts) were collected and measured in absorbance of 260 nm. Lysates were layered over 10–50% or 15–50% of cold sucrose gradients in buffer (10 mM HEPES-KOH at pH 7.4, 2.5 mM MgCl_2_, and 100 mM KCl). Gradients were centrifuged at 17,000 rpm in a Beckman SW28 rotor for 15 or 13.5 h at 4°C. After centrifugation, 12 equal-sized fractions (1.2 ml/fraction) were collected and the absorbance was measured.

## Results

### Sal in Combination With 4E1RCat Synergistically Decreased Melanoma Cell Viability

**Figure 1:**
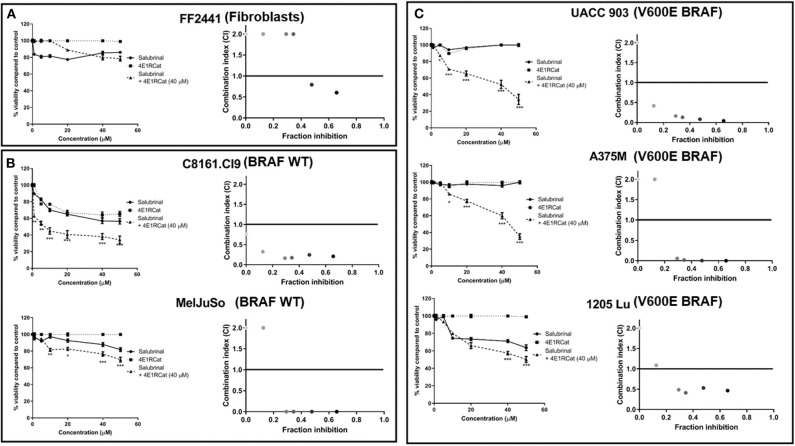
Combination of Salubrinal and 4E1RCat synergistically decreased the viability of melanoma cell line containing or lacking BRAF V600E. Normal human fibroblast **(A)**, BRAF wild-type melanoma **(B)**, and BRAF-mutant melanoma **(C)** cell lines were treated with 40 μM 4E1RCat, 0–50 μM Sal, 4E1RCat/Sal combination, or DMSO control, and cell viability was measured by MTS assay. Points; average, ± SEM. *p < 0.05, **p < 0.01, ***p < 0.001. CalcuSyn analysis identified that 40 μM 4E1RCat and 10–50 μM Sal were synergistic in all melanoma cell lines compared to the individual agents.

Knockdown of ALDH18A1 protein levels has been shown to significantly decrease melanoma cell viability and tumor growth, leading to eIF2α phosphorylation through GCN2 activation (12). Since no drugs are available that target alDH18A1, chemical agents disrupting eIF2α or the eIF4F complex were investigated as an approach to target this pathway. Treatment of melanoma cell lines with either Sal or 4E1RCat alone had a minimal effect on cell viability. In contrast, the combination treatment was surprisingly effective at decreasing the viability of cancer compared to normal cells (Figure 1). The Sal/4E1RCat combination had a minimal effect on the normal non-cancerous FF2441 fibroblast cell line (Figure 1A), but statistically significantly decreased viability in melanoma cell lines containing mutant BRAF V600E and those lacking the mutant BRAF protein (Figures 1B,C). To determine if the combination effect on cell viability was additive or synergistic, CI values using the Chou-Talalay method were assessed. The combination of 40 μM 4E1RCat with a range of Sal combinations from 10 to 50 μM was determined to be highly synergistic in all melanoma cell lines tested as compared with either drug alone (Figure 1). Since all cell lines, irrespective of BRAF mutational status, demonstrated highly synergistic inhibition when treated with the drug combination, it appears that this treatment approach could work with BRAF wild-type and mutant cell lines. Subsequent studies therefore were undertaken primarily on the UACC 903 and/or 1,205 Lu cell lines with additional lines included to emphasize a particular result.

### Combination Sal and 4E1RCat Decreased Melanoma Xenograft Tumor Growth

**Figure 2:**
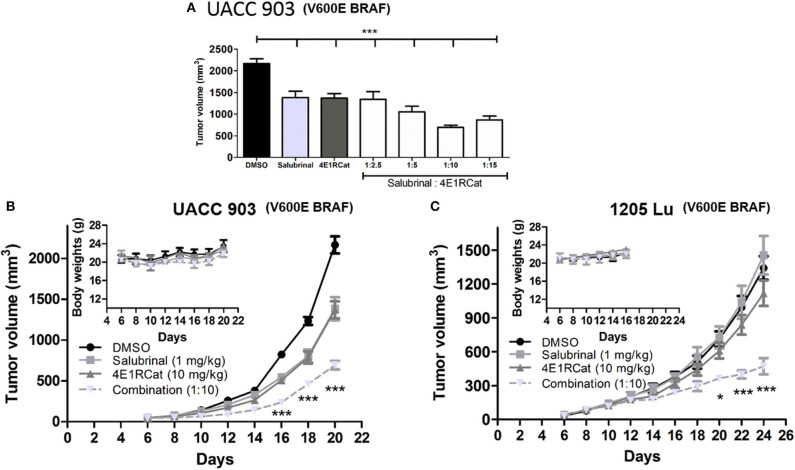
Salubrinal and 4E1RCat combination significantly decreased melanoma tumor development. **(A)** UACC 903 cells subcutaneously injected into nude mice were injected intraperitoneally (IP) with indicated concentrations of Sal, 4E1RCat, and the combination. Twenty days after injection, tumor volume was assessed. Bars; average, ± SEM. **(B,C)** UACC 903 **(B)** or 1,205 Lu **(C)** cells were subcutaneously injected into nude mice followed by IP injection of indicated concentrations of Sal, 4E1RCat, and combination. Tumor volumes were measured on alternate days (*N* = 8). Points; mean, ± SEM (statistics, one-way ANOVA followed by Tukey's test for multiple comparisons). No significant body weight difference was observed, indicating negligible toxicity (inset). *p < 0.05, ***p < 0.001 of the drug combination to the single agents or vehicle-treated controls.

To determine the optimal ratio of Sal and 4E1RCat for *in vivo* treatment, a range of concentrations of 4E1RCat from 2.5 to 15 mg/kg body weight were combined with 1 mg/kg Sal and administered intraperitoneally to UACC 903 xenograft mice. The combination of 1 mg/kg Sal and 10 mg/kg 4E1RCat was found to impair tumor growth greatest after 20 days (Figure 2A), and was thus chosen for further investigation. Similar results in the UACC 903 and 1,205 Lu cell lines confirmed that the observed results were not specific to a particular cell line and that the 1:10 mg/kg combination significantly impaired xenograft tumor growth greater than either drug alone (Figures 2B,C). Furthermore, it did not significantly alter mouse body weight, which suggested negligible toxicity (Figures 2B,C, insets). Measurement of serum parameters from mice treated with 1 mg/kg Sal and 15 mg/kg 4E1RCat identified that levels of CAL and total protein (TP) were slightly below DMSO control levels, outside of the normal range (Table 1), which was expected since protein production was targeted.

**Table 1:** Serum analysis of highest dose combination reveals no significant organ-related toxicity.

**Serum parameter**	**Standard range**	**Vehicle**	**Salubrinal**	**4E1RCat**	**Combination**
**Dose**			**1 mg/kg**	**15 mg/kg**	
**GLU**	**198–232 mg/dl**	178.3 ± 42.19	193.3 ± 6.94	214 ± 12.5	169 ± 2.51
**BUN**	**18–33.7 mg/dl**	22 ± 5.51	29.6 ± 2.33	30.3 ± 3.18	24.3 ± 4.48
**CREA**	**0–0.31 mg/dl**	0.13 ± 0.03	0.13 ± 0.33	0.13 ± 0.03	0.16 ± 0.03
**CAL**	**7.1–10.1 mg/dl**	8.83 ± 1.71	10.8 ± 0.11	10.7 ± 0.06	5.5 ± 1.25
**TP**	**3.5–7.2 g/dl**	4 ± 1.05	5.1 ± 0.17	5.26 ± 0.12	2.66 ± 0.59
**ALB**	**2.5–4.8 g/dl**	2.23 ± 0.16	2.56 ± 0.08	2.63 ± 0.13	2.13 ± 0.65
**GLOB**	**2.5–4.6 mg/dl**	2.3 ± 0.36	2.4 ± 0.14	2.66 ± 0.06	2.23 ± 0.47
**ALT**	**33–132 U/L**	74.3 ± 19.6	55.6 ± 4.37	54.3 ± 4.05	73.3 ± 17.69
**ALKP**	**62–209 U/L**	68.3 ± 7.69	57.3 ± 7.89	76.3 ± 7.54	67.2 ± 27.65
**TBIL**	**0.2–0.9 mg/dl**	0.56 ± 0.27	0.53 ± 0.14	0.46 ± 0.03	0.23 ± 0.08
**AMYL**	**1,200–2,000 mg/dl**	*1, 917*±280	*2, 110*±201	*1, 712*±260	*1, 599*±348

*Blood parameters of nude mice from Figure 2A treated with DMSO, 1 mg/kg Sal, 15 mg/kg 4E1RCat, or 1:15 mg/kg Sal/4E1RCat. ALT, Alanine aminotransferase; ALKP, alkaline phosphatase; ALB, albumin; GLOB, globulin; TP, total protein; TBIL, total bilirubin; BUN, blood urea nitrogen; GLU, glucose; CREA, creatinine; AMYL, amylase; CL, calcium. Bold values represent standard range for the tests*.

### Sal and 4E1RCat Impaired Cell Cycle Progression and, in One Melanoma Cell Line, Clearly Inhibited Protein Synthesis

**Figure 3:**
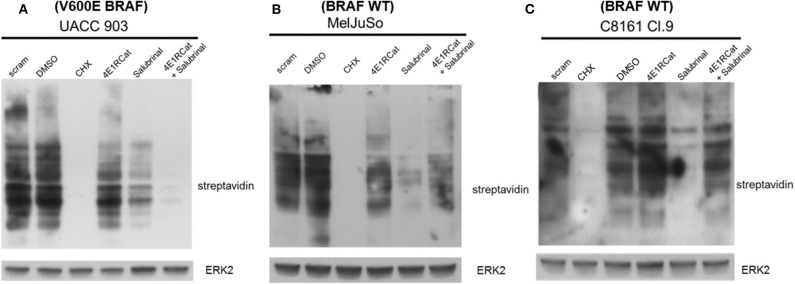
Salubrinal and 4E1RCat decreased protein synthesis. UACC 903 **(A)**, MelJuSo **(B)**, and C8161.Cl9 **(C)** cells were incubated with 10 μM 4E1RCat, 40 μM Sal, or 4E1RCat/Sal combination for 2 days, followed by methionine starvation and subsequent azidohomoalanine (AHA) incubation for 4 h. Cycloheximide (CHX) served as a positive assay control. Biotin-alkyne was attached to newly synthesized proteins containing AHA followed by Western blot analysis. Membranes were probed with streptavidin-HRP to identify newly synthesized proteins. ERK2 primary antibody served as a control for equal protein loading.

The next goal was to determine the mechanism by which Sal and 4E1RCat impaired melanoma cell viability and xenograft tumor growth. Since both drugs are known to inhibit processes involved in protein synthesis, the methionine analog azidohomoalanine (AHA) was used to measure the extent of new protein synthesis (34). In UACC 903 cells, the combination of Sal and 4E1RCat inhibited protein synthesis greater than either drug alone (Figure 3A). However, in the MelJuSo and C8161.Cl9 cell lines (Figures 3B,C), the combination was also able to decrease protein synthesis as compared with untreated and DMSO controls. It is not clear why Sal treatment alone inhibited protein synthesis greatest, which possibly induced feedback to block this process when the drugs were combined. The growth inhibitor effect was speculated to be caused by the lack of production of particular proteins such as those involved in the cell cycle and cell cycle progression.

**Figure 4:**
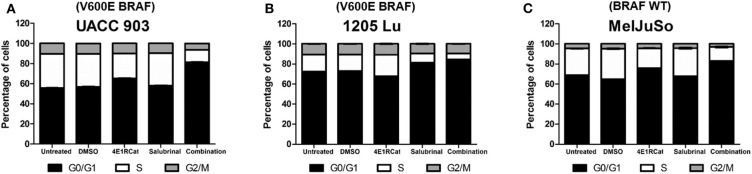
Salubrinal and 4E1RCat induced cell cycle arrest. UACC 903 **(A)**, 1205 Lu **(B)**, and MelJuSo **(C)** cells were incubated with 10 μM 4E1RCat, 40 μM Sal, or 4E1RCat/Sal combination for 2 days followed by staining with propidium iodide, analysis with BD FACSCalibur, and data analysis using ModFit LT (*N* = 3).

**Figure 5:**
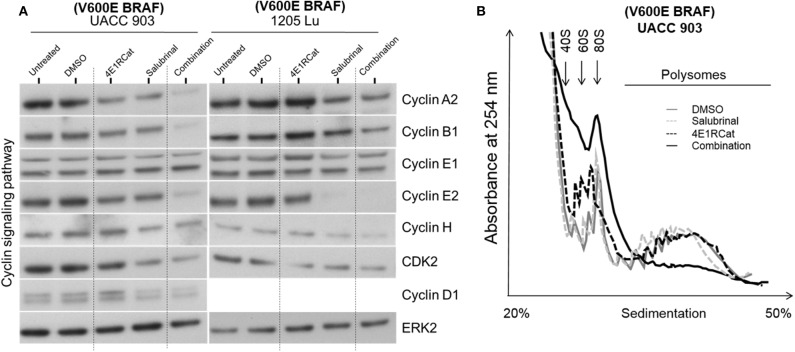
Salubrinal and 4E1RCat combination decreased protein expression of cyclins and impaired polysome assembly to inhibit protein translation. **(A)** UACC 903 and 1,205 Lu cells were treated with 10 μM 4E1RCat, 40 μM Sal, or 4E1RCat/Sal combination for 2 days. Western blots were probed for total protein levels of multiple cyclins. ERK2 was used as a protein loading control. **(B)** UACC 903 cells were treated with 10 μM 4E1RCat, 40 μM Sal, or 4E1RCat/Sal combination for 24 h followed by addition of CHX for 10 min. Cell lysates were ultracentrifuged on sucrose gradients and the ribosomal/polysomal fractions were evaluated.

To determine if alterations in cell cycle progression and particular protein involved in this process may have affected melanoma cell viability and tumor growth, propidium iodide was used to measure DNA levels following treatment. In all melanoma cell lines tested, the combination of Sal and 4E1RCat decreased the percentage of cells in S phase and increased the percentage of cells in G0/G1 phase greater than either drug alone (Figure 4). This observation again suggested that the effect of the drug combination was similar irrespective of the BRAF mutational status, suggesting the possible regulation of protein production of those involved in the cell cycle. This possibility was examined by determining whether changes in expression of several proteins involved in cell cycle progression may have contributed to cell cycle arrest. In UACC 903 and 1205 Lu cells, combination treatment decreased protein levels of cyclin A2, cyclin B1, cyclin D1, cyclin E2, cyclin H, and Cdk2 greater than or equal to treatment of either drug alone (Figure 5A). Only cyclin E1 protein levels were unchanged between treatment groups and controls (Figure 5A). Cyclin D1 and several of these proteins have been implicated in disruption of the cell cycle or cell cycle arrest (35, 36).

Since Sal and 4E1RCat impaired cell cycle progression and cyclin expression, polysome analysis was performed to determine whether the drug combination impaired protein translation. In UACC 903 cells, the combination of Sal and 4E1RCat decreased the polysome fraction greater than either drug alone with an increase in the 40S, 60S, and 80S ribosomal fraction indicating the inhibition of protein translation (Figure 5B).

## Discussion

The American Cancer Society has reported that the number of new melanoma cases has increased by over 50% in the past decade in the United States (37, 38). Although immunotherapies and combination BRAF and MEK inhibitor treatments have improved survival rates, many patients still relapse (39–41). As the protein synthesis machinery has been reported as an important component downstream of signaling cascades disrupted in melanoma (11), the identification of agents to target this pathway could assist in overcoming resistance. Previous work identified P5CS as a potential target with inhibition disrupting activated GCN2, impairing eIF2 function, and decreasing protein synthesis in melanoma (12). Since inhibitors of P5CS have not been reported, this report determined whether impairing eIF2 function and pathway signaling could be a useful therapeutic approach.

Sal is a drug that impairs eIF2α dephosphorylation (42). Studies have reported that Sal increased sensitivity of cancer cell and xenograft tumor models to chemotherapy or rapamycin (28, 43, 44). 4E1RCat was identified as a molecule capable of impairing eIF4 assembly (20). Although 4E1RCat has not been extensively studied, other inhibitors of eIF4F assembly members such as 4EGI-1 and silvestrol have been reported to inhibit melanoma growth (45–47). This report has identified Sal and 4E1RCat as two molecules that together are capable of disrupting melanoma viability. The effect occurred in all cell lines having or lacking mutant BRAF V600E but, importantly, did not significantly alter normal fibroblast cell viability. As oncogenic signaling pathways activated in melanoma converge on the protein synthesis machinery, melanoma may be more sensitive to perturbation than normal cells and the effect was not restricted to cells containing mutant active BRAF V600E but also occurred with cell lines lacking the mutation. These cell lines activate the MAP kinase pathway through upstream modifications in the signaling cascade supporting the possibility that oncogenic signaling pathways activated in melanoma converge on the protein synthesis machinery (48).

Since protein synthesis is required for normal cell activity but at lower levels than required by cancer cells, it is important to examine the possible side effects of targeting protein synthesis machinery. Historically, protein synthesis inhibitors targeting the elongation step of mRNA translation produced non-specific toxicity that limited clinical value (49). However, it is becoming apparent that inhibitors targeting translation initiation may be potentially useful for treating cancer (50). The differential selection of mRNAs for translation by the initiation complex could explain the more favorable response, whereby genes involved in growth, survival, cell cycle, and apoptosis have higher translation rates in conditions of increased eIF4F levels (51–53). In support of the notion that targeting translation initiation may be less toxic, no overt toxicity was observed following combination treatment, as demonstrated by mouse body weight but several serum markers of organ toxicity were outside of the normal range. Specifically, TP levels were lower in the combination treatment than observed with either drug alone. This may be expected as these agents impair protein synthesis.

Importantly, treatment of each melanoma cell line with either drug alone or the combination decreased TP synthesis or those specifically involved in cell cycle regulation as compared with untreated and DMSO-treated controls. However, considering that the combination treatment synergistically decreased cell viability, it was unexpected to see that in two of the three cell lines tested, the combination treatment did not impair TP synthesis additively or synergistically. It is possible that this combination could be inducing a synergistic effect on cell viability by disproportionately impairing the synthesis of proteins involved in oncogenic activities, particularly those of the cell cycle, more so than affecting overall protein synthesis. This is supported by the results showing that the combination induced cell cycle arrest and decreased expression of several cyclins greater than either drug alone. Furthermore, future studies could examine the mechanistic contribution of either drug to the synergistic effect observed in melanoma cell viability by targeting various downstream components, such as by using ISRIB to impair the effect of sustained eIF2α phosphorylation (54, 55).

In conclusion, this report demonstrates that the combination of Sal and 4E1RCat synergistically impairs melanoma development, cell cycle progression, and expression of cyclins. This is important since the single drugs were not as effective as the combined agents. Therefore, under circumstances in cancer where the single agents are not effective at inhibiting protein production, the Sal and 4E1RCat combination could be used. Importantly, this combination had no effect on normal fibroblast cells and had negligible toxicity in mice. Thus, this study suggests that the protein translational machinery may be an effective target for melanoma with no effects on normal cells and manageable toxicity in animals. It will be of interest to elucidate whether this treatment can overcome BRAF and MEK inhibitor resistance, or if it could synergize with current treatment options.

## Data Availability Statement

The datasets generated for this study are available on request to the corresponding author.

## Ethics Statement

The animal study was reviewed and approved by IACUC Committee, Penn State College of Medicine, Hershey, PA, 17033.

## Author Contributions

GK, RG, SD, SK, and GR contributed for conception and design of the study. GK, RG, and SD performed the experiments. SK and GR contributed the resources, lab space and equipment. GK wrote the first draft of the manuscript. RG, SD, SK, and GR wrote sections of the manuscript. All authors contributed to manuscript revision, read, and approved the submitted version.

## Conflict of Interest

The authors declare that the research was conducted in the absence of any commercial or financial relationships that could be construed as a potential conflict of interest.
